# Smartphone applications are used for self-management, telerehabilitation, evaluation and data collection in low back pain healthcare: a scoping review

**DOI:** 10.12688/f1000research.123331.2

**Published:** 2024-05-07

**Authors:** Lech Dobija, Jean-Baptiste Lechauve, Didier Mbony-Irankunda, Anne Plan-Paquet, Arnaud Dupeyron, Emmanuel Coudeyre

**Affiliations:** 1 UNH, INRAE, Université Clermont-Auvergne, Clermont-Ferrand, Puy de Dôme, 63000, France; 2 Service de Médecine Physique et de Réadaptation, Centre Hospitalo-Universitaire (CHU) de Clermont Ferrand, Cébazat, Puy de Dôme, 63118, France; 3 Université Montpellier, Nimes, 30900, France; 4 Service de Médecine Physique et de Réadaptation, Centre Hospitalo-Universitaire (CHU) de Nimes, Nimes, 30900, France

**Keywords:** Smartphone apps, low back pain, mHealth, self-management, telerehabilitation

## Abstract

**Background:**

Smartphone use has grown in providing healthcare for patients with low back pain (LBP), but the literature lacks an analysis of the use of smartphone apps. This scoping review aimed to identify current areas of smartphone apps use for managing LBP. We also aimed to evaluate the current status of the effectiveness or scientific validity of such use and determine perspectives for their potential development.

**Methods:**

We searched PubMed, PEDro and Embase for articles published in English up to May 3 ^rd^, 2021 that investigated smartphone use for LBP healthcare and their purpose. All types of study design were accepted. Studies concerning telemedicine or telerehabilitation but without use of a smartphone were not included. The same search strategy was performed by two researchers independently and a third researcher validated the synthesis of the included studies.

**Results:**

We included 43 articles: randomised controlled trials (RCTs) (n=12), study protocols (n=6), reliability/validity studies (n=6), systematic reviews (n=7), cohort studies (n=4), qualitative studies (n=6), and case series (n=1). The purposes of the smartphone app were for 1) evaluation, 2) telerehabilitation, 3) self-management, and 4) data collection. Self-management was the most-studied use, showing promising results derived from moderate- to good-quality RCTs for patients with chronic LBP and patients after spinal surgery. Promising results exist regarding evaluation and data collection use and contradictory results regarding measurement use.

**Conclusions:**

This scoping review revealed a notable interest in the scientific literatures regarding the use of smartphone apps for LBP patients. The identified purposes point to current scientific status and perspectives for further studies including RCTs and systematic reviews targeting specific usage.

## Introduction

Technological development has accelerated notably in the field of telecommunication in the last few decades. Since the introduction of the first mobile phones, the number of users has continued to grow and is now estimated at 6.8 billion worldwide. ^1^ Mobile phones have gained new capabilities such as better Internet connection, allowing them to substitute for personal computers to a large extent. This new generation of mobile phones, called smartphones, has changed a lot of human activity. The number of smartphone users worldwide has surpassed three billion and is forecast to further grow. ^2^ Extensive software of smartphones has permitted the development of new applications, allowing video communication, social media consulting, gaming, and navigation. The potentially inexhaustible use of the smartphones cannot be ignored in any human activity including healthcare.

The dynamic technological development has been accompanied by a less spectacular increase in life expectancy. However, simultaneously, the rate of years living with disability (YLD) has stagnated or even increased for some diseases. The last estimations of the YLD positioned low back pain (LBP) as a leading cause of handicap worldwide. ^3^ Besides the obvious healthcare problem, the economic impact of LBP is serious, with the total mean cost per patient over six months estimated at EUR 715.6 in France. ^4^ One of the aspects of this problem is that LBP could be chronic or recurrent in nature and affects middle-aged, professionally active adults. Chronic LBP concerns less than 10% of cases but 85% of the costs. ^5^ A further problem is that in most cases, physicians are unable to reliably identify the cause of the LBP symptoms, so they classify them as non-specific.

When nonspecific LBP persists and becomes chronic, it becomes increasingly complex and challenging to manage due to its multifactorial nature. ^6^ Failure of the purely biomedical approaches targeting only pathoanatomical nociceptive aspects has led to the development of the more exhaustive bio-psycho-social model. This multidimensional approach encompasses psychological, biological, social, and environmental aspects. ^7^ Current guidelines for management of chronic LBP include psychosocial interventions along with exercise therapy, medications, multidisciplinary rehabilitation and spinal manipulation. ^8^ Completing the context of LBP, some authors highlight the problem of overtreating. Expanding testing and treatment by using therapies and diagnostic tools with weak scientific validity can drive increasing complication rates, marketing abuse and patients’ confusion. ^9^ The widespread occurrence of LBP self-management strategies seems appropriate to target economic and healthcare accessibility problems. ^10^

Smartphone applications (apps) for this purpose appear to be promising tools, giving a wide range of possibilities for use, replacing education booklets, proposing and supervising exercise therapy (telerehabilitation), and stimulating adherence for self-management programs. Using smartphone apps are the most accessible way to provide rehabilitation services and to collect outcomes remotely as smartphones are personal and always available to patients. ^11^^,^^12^ Such a new model makes health services more accessible and enhances patient participation and their engagement in self-management. ^11^ Compared to face-to-face treatment smartphone apps could potentially be more cost-effective and could help to overcome the problem of healthcare accessibility. This idea has been developed in other healthcare intents such as diabetes, ^13^ chronic obstructive pulmonary disease, ^14^ and osteoarthritis. ^15^ However, smartphones equipped with multiple sensors, cameras, gyroscopes, accelerometers, and magnetometers could also be used as tools for range-of-motion (ROM) measurement ^16^ or could be simply used with the phone camera for clinical evaluation at a distance. ^17^ Another feature is that smartphone apps could be used for surveying large population samples, allowing to create a database for more sophisticated analyses including case-based reasoning systems. ^18^ Moreover, the need for healthcare management at a distance (telemedicine) has become crucial in the recent situation requiring confinement due to the coronavirus disease 2019 (COVID-19) pandemic. Without the possibility to access conventional healthcare face-to-face with providers, numerous clinicians were challenged to provide telemedicine in order to substitute conventional healthcare in non-essential services. ^19^^,^^20^ Smartphone applications have become highly pertinent tools for this purpose.

Indeed, the use of smartphone apps has increased for LBP patients. However, there is a lack of synthesis of the scientific literature in the areas of smartphone app use. Moreover, it is not clear in what purpose using smartphone apps are pertinent and if such use is supported by the scientific studies. Giving a large range of area of interest and the fact that it is a new dynamically developing subject we believe that performing a scoping review will clarify these questions.

The objective of this scoping review is to identify current areas of smartphone app use for managing LBP. It also aimed to evaluate the current status of the effectiveness or scientific validity of smartphone app use and to determine perspectives for their potential development.

## Methods

### Protocol and registration

Review protocol presenting search strategy was established without a registration number.

### Information sources and search strategy

The Preferred Reporting Items for Systematic reviews and Meta-Analyses extension for Scoping Reviews (PRISMA-ScR) checklist was used to guide the present study. ^21^^,^^63^ Two researchers (LD and JBL) independently searched for articles in PubMed (RRID:SCR_004846), PEDro and EMBASE (RRID:SCR_001650) by using MeSH keywords. The search strategy phrase for PubMed database was (“smartphone application” OR “smartphone app” OR “smartphone” OR “telerehabilitation” OR “telemedicine” OR “mhealth” OR “ehealth”) AND (“low back pain” OR “back pain”). Corresponding research was realized in PEDro and EMBASE by using the same keywords. Only English language and time frame filters were used for our research. The articles were screened and assessed for eligibility regarding the objective of the study. Articles published between January 1st 2005 and May 3rd 2021 were considered. This time frame includes the emergence of the new generation of mobile phones.

### Eligibility criteria

Only studies of smartphone use in LBP adult patient healthcare were included. We excluded studies concerning telemedicine or telerehabilitation but without use of a smartphone. Similarly, we excluded studies in which the smartphone use involved healthy individuals for preventing LBP or promoting physical activity or a healthy lifestyle in healthy individuals. To broaden our review, all qualitative and quantitative studies were accepted, including randomised controlled trials (RCTs) and non-randomized studies, cohort studies, case-control studies, systematic reviews, reliability and validity studies, and study protocols. In cases where both the protocol and the completed study article were published, we included both to ensure comprehensive content analysis of the literature on the subject. Only studies accepted for publication, written in English were considered.

### Selection of sources of evidence

Study inclusion was discussed to reach agreement or in cases where a consensus could not be reached, we consulted with a third researcher (EC). Then studies were classified according to the purpose of the smartphone app. The type of study design was also used to classify studies in terms of its objective: effectiveness of the smartphone use, reliability of the smartphone measures, or other type of evaluation and data collection.

### Data charting process

Search strategy was prepared and validated with the participation of all authors of the present review. The same search strategy was realized by two researchers (LD and JBL) independently. A third researcher (EC) participated in the synthesis data charting. Then, additional articles were identified throughout citation matching.

### Data items

We extracted data concerning smartphone app utilisation. We focused on the purpose of smartphone app utilisation, study design, date of publication, number of participants who completed the study, main outcomes, results and authors conclusions.

### Critical appraisal of individual sources of evidence

The quality of selected RCTs was estimated by using the PEDro scale. ^22^

### Synthesis of results

We categorized included studies by the purpose of smartphone app utilisation. The synthesis of the included studies was also done by study design, both were presented in a narrative format and in a synthesis table.

## Results

**Figure 1.**
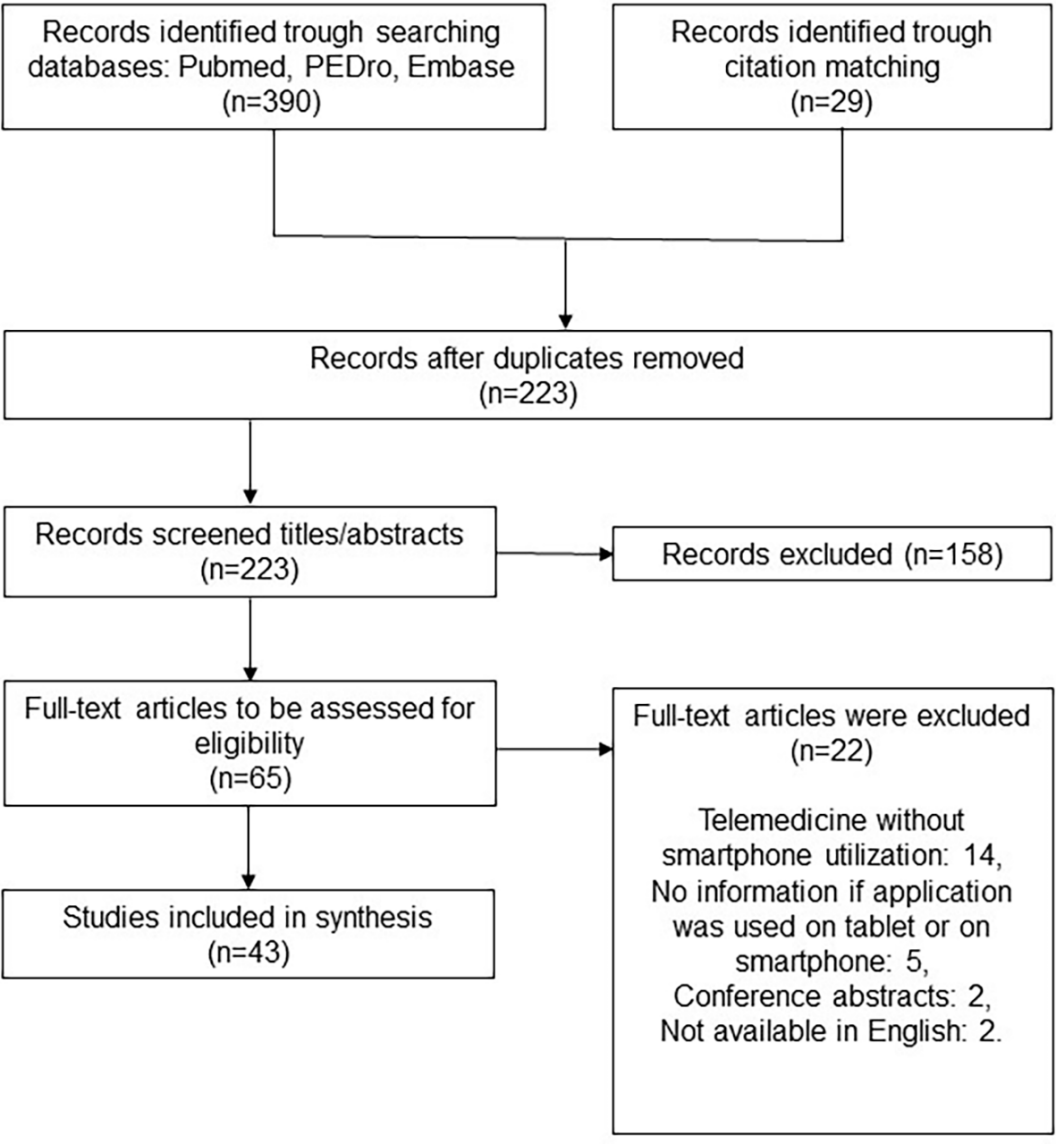
Flow diagram of studies selection.

We identified 43 articles based on our search strategy ( Figure 1). The included studies were RCTs (n=12), study protocols (n=6), reliability/validity studies (n=6), systematic reviews (n=7), cohort studies (n=4), qualitative studies (n=6), and case series (n=1) ( Table 1).

**Table 1.** List of articles included in the scoping review.

Author, publication date (reference number)	Study design	Objective	Purpose of the smartphone use
**Nordstoga, 2020** ^56^	Qualitative feasibility and quantitative pilot study	To assess the usability and acceptability of the *selfBACK* smartphone app.	SM, DC
**Grolier, 2020** ^55^	Mixed quantitative and qualitative approaches	To refine the design of a smartphone application for people with chronic non-specific LBP.	SM
**Almhdawi, 2020** ^58^	Pilot RCT	To evaluate the effectiveness of a newly developed evidence-based LBP management using smartphone app on pain and disability.	SM
**Pourahmadi, 2020** ^30^	Systematic review	To evaluate available evidence in the literature to assess the psychometric properties of the iHandy Level app in measuring lumbar spine ROM and lordosis.	E
**Coe-O'Brien, 2020** ^33^	Systematic scoping review	To assess the quality of the free Smartphone Apps for LBP; outcome measures used; the outcome measures against the International Classification of Functioning, Disability and Health core set classifications for LBP.	SM, E
**Sandal, 2019** ^50^	Protocol of RCT	To evaluate the effectiveness of using the *selfBACK* app to support self-management in addition to usual care *versus* usual care only in people with non-specific LBP on pain-related disability.	SM, DC
**Pourahmadi, 2019** ^27^	Reliability study	To evaluate the reliability and validity of an application (iHandy® Level) for measuring active lumbar flexion-extension ROM in chronic non-specific LBP patients.	E
**Pourahmadi, 2019** ^16^	Systematic review	To evaluate available evidence in the literature to assess the psychometric properties of the iHandy Level app in measuring lumbar spine ROM and lordosis.	E
**Riis, 2018** ^53^	Qualitative study	To identify preferences for the content, design, and functionality of a Web app with evidence-based information and advice for people with LBP.	SM
**Machado, 2016** ^10^	Systematic review of the LBP apps	To screen app stores for the self-management of LBP apps and evaluate their content quality and whether they recommend evidence-based interventions.	SM
**Dario, 2017** ^40^	Systematic review with meta-analysis	To evaluate whether interventions delivered by telehealth improve pain, disability, function, and quality of life in non-specific LBP.	TR
**Farasyn, 2013** ^34^	Reliability, validity study with RCT design	To explore the reliability, responsiveness and concurrent validity of the BADIX score using an app.	E
**Stienen, 2019** ^31^	Validity study	To compare 6-minute walking distance measurements obtained with a newly developed smartphone application and those obtained with the gold-standard distance wheel.	E
**Mork, 2018** ^18^	Study protocol	To implement a Decision Support System for self-management of non-specific LBP, utilizing smartphone app to develop personalized self-management plans and evaluate its effect.	SM
**Verbrugghe, 2017** ^39^	Qualitative study	To investigate inventory training preferences and motives, evaluate whether these change during rehabilitation, and evaluate familiarity with using technologies, in persons with non-specific LBP.	SM, TR
**Blödt, 2014** ^51^	Study protocol	To evaluate whether an additional app-delivered relaxation is more effective in the reduction of chronic LBP than usual care alone.	SM
**Mbada, 2019** ^32^	Randomized trial	To compare the effects of Telerehabilitation-Based McKenzie Therapy and Clinic-Based McKenzie Therapy among patients with LBP.	E, TR
**Hou, 2019** ^38^	RCT	To evaluate the effectiveness of mobile phone-based rehabilitation systems on pain and disability in patients who underwent lumbar spinal surgery.	TR, SM
**Peterson, 2018** ^37^	Case series	To describe the implementation of Telerehabilitation booster sessions and remote patient monitoring in three patients with chronic LBP.	TR, SM
**Peterson, 2019** ^17^	Validity study	To evaluate the agreement between telerehabilitation and face-to-face assessments of patients with acute and subacute LBP using a modified treatment-based classification system, and patient satisfaction.	E, TR
**Amorim, 2016** ^45^	Protocol of RCT	To investigate the effectiveness of a mobile health supported physical activity intervention in care-seeking, pain and disability in people with chronic LBP after discharge from treatment.	TR, SM
**Amorim, 2019** ^36^	RCT pilot	To evaluate the feasibility and preliminary effectiveness of a patient-centred physical activity intervention, supported by health coaching and mobile health, to reduce care-seeking, pain and disability in patients with chronic LBP.	TR, SM
**Chhabra, 2018** ^11^	RCT	To evaluate the effect of using a smartphone app on pain and function in patients suffering from chronic LBP.	SM, DC
**Jamison, 2019** ^25^	RCT	To examine the benefit of a high-frequency transcutaneous electrical nerve stimulation device for patients with chronic LBP.	E, DC
**Lin, 2019** ^24^	Pilot study, clinical trail	To evaluate the feasibility and usability of an ecological momentary pain assessment with a smartphone application.	E, DC
**Lee, 2016** ^28^	RCT	To evaluate the effects of the Graston technique and general exercise on pain and range of motion in patients with chronic LBP.	E
**Ross, 2020** ^26^	Retrospective cohort study	To identify factors that predicted the benefits and future use of a smartphone pain app among patients with chronic pain.	SM, E, DC
**Hasenöhrl, 2020** ^35^	Qualitative feasibility and quantitative pilot study	To evaluate the feasibility and acceptance of orthopaedists prescribing individualized therapeutic exercises *via* a smartphone app to patients suffering from non-specific LBP.	TR, SM
**Corrêa, 2020** ^23^	Reliability, validity study	To test the inter- and intra-rater reliabilities and concurrent validity of smartphone app for quantification of pain drawings in patients with LBP.	DC, E
**Yeh, 2020** ^59^	Study protocol of RCT	To test auricular point acupressure as a non-invasive, non-pharmacological self-management strategy to manage chronic LBP.	DC
**O' Halloran, 2019** ^52^	Study protocol of RCT	To describe the design of a cluster RCT evaluating the effect of Motivational Interviewing combined with usual physiotherapy care and a specifically designed app to increase physical activity in people with LBP.	SM
**Carpenter, 2019** ^60^	Cohort study	To evaluate reciprocal associations between opioid use and physical pain using ecological momentary assessment *via* a smartphone app.	DC
**de Brito Macedo, 2020** ^29^	Reliability, validity study	To evaluate the concurrent validity and the intra-rater reliability of thoracolumbar range of motion measurement using a mobile application and a digital inclinometer in individuals with and without back pain.	E
**Yang, 2019** ^57^	RCT - pilot study	To evaluate the additional effect of self-management on physiotherapy *via* the use of smartphone app on management of chronic LBP.	SM
**Du, 2019** ^46^	Systematic review & meta-analysis	To evaluate the effectiveness on pain intensity and disability of eHealth based self-management programs on chronic LBP.	SM
**Suman, 2019** ^48^	Cluster RCT	To evaluate the effectiveness and cost-utility of a multifaceted eHealth strategy compared to usual care in improving patients' back pain beliefs, and in decreasing disability and absenteeism.	SM
**Selter, 2018** ^44^	Clinical trial - pilot study	To examine patient engagement, patient-perceived utility with the smartphone app-based self-management program and assess the validity of the Your Activities of Daily Living module for quantifying functional status among patients with chronic LBP.	SM, TR
**Clement, 2018** ^42^	Retrospective cohort study	To elucidate the effect on user retention and clinical outcomes of an updated version of the *Kaia* app.	DC, SM, TR
**Toelle, 2019** ^41^	RCT	To evaluate the clinical effects of a multidisciplinary mHealth back pain App ( *Kaia* App) in an RCT.	SM, TR
**Huber, 2017** ^43^	Retrospective cohort study	To report on the retrospective short-term results of a digital multidisciplinary pain app ( *Kaia* App) for the treatment of LBP.	DC, SM, TR
**Ganguli, 2017** ^54^	Qualitative study	To facilitate local video creation to deliver targeted information to patients.	SM, DC
**Nicholl, 2017** ^47^	Systematic review	To review the use of interactive digital interventions to support self-management of LBP.	SM
**Irvine, 2015** ^49^	RCT	To evaluate the effectiveness of a mobile-Web intervention called *FitBack* on physical, behavioral, and worksite outcomes, to help users implement self-tailored strategies to manage and prevent LBP.	SM

SM, self-management; TR, telerehabilitation; DC, data collection; E, evaluation; RCT, randomized controlled trail; LBP, low back pain; ROM, range of motion.

We identified the following purposes of the smartphone apps: 1) evaluation, 2) telerehabilitation, 3) self-management or 4) data collection. We distinguished telerehabilitation and self-management because of a difference when the remote interaction between healthcare professional and patients occurred for LBP management (telerehabilitation). By contrast, some smartphone apps provide instructions for LBP management without giving feedback from a healthcare professional (self-management). Furthermore, the data collection use in most smartphone apps was coupled with other employment-like evaluations or self-management, but in some studies, the smartphone app was mainly dedicated to collecting information for further analysis.

### Use of smartphone apps for evaluation

A total of 14 studies used smartphone apps as an evaluation tool including pain evaluation, ^23^^–^^26^ ROM measurement, ^16^^,^^27^^–^^30^ 6-min walk test ^31^ and clinical or functional evaluation. ^17^^,^^32^^–^^34^ Considering pain evaluation, PainMAP showed excellent intra- and inter-rater reliability and good validity for quantifying the number of pain sites and pain area. ^23^ This application allows patients to mark their pain on digital body charts and automatically quantifies such self-assessment. Furthermore, another smartphone app could effectively evaluate pain changes after transcutaneous electrical nerve stimulation. ^25^ However, Ross *et al.*, reported that patients with chronic pain who appeared to manage their pain better were less likely to report benefits of a smartphone pain app designed for daily pain management and evaluation. ^26^

Smartphone apps were used to measure lumbar spine ROM and lordosis but showed insufficient reliability and validity as compared with a gravity-based inclinometer. ^27^ By contrast, de Brito Macedo *et al.*, reported good concurrent validity and intra-rater reliability of smartphone thoracolumbar ROM measurements *versus* a digital inclinometer. ^29^ Lumbar ROM measurement with a smartphone detected a significant difference due to manual therapy and exercise intervention in another study. ^28^ Similar findings were further summarized in systematic review showing contradictory results of psychometric properties of the lumbar spine ROM and lordosis measurements with a smartphone app. ^30^

Other examples of smartphone use included clinical and functional evaluation. Peterson *et al.*, suggested that a modified treatment-based classification system for subgrouping patients with LBP could direct treatment in telerehabilitation settings (smartphone app) because the overall rate of percentage agreement with face-to-face assessments was between 48.9% and 59.6%. ^17^ The McKenzie classification and therapy applied with a smartphone app showed comparable clinical outcomes with the traditional clinic-based McKenzie therapy. ^32^ The 6-min walk test performed with a smartphone app and using a GPS system could be reliable but needed to be performed avoiding indoors and city environments with high buildings and rectangular walking curses. ^31^ However, in a scoping review of outcomes with smartphone apps used for LBP management, authors showed the problem of low quality of the outcome measures to monitor the treatment effect. Indeed, only a few smartphone apps offered to monitor their effectiveness in the management of LBP. ^33^ Other examples of smartphone use for clinical evaluation is the use of the Backache Disability Index. Such evaluation includes rating five trunk movements in the erect position and scoring morning back stiffness. Performed remotely with a smartphone app, the index showed good reliability and validity. ^34^

### Use of smartphone apps for telerehabilitation

A total of 12 studies presented smartphone apps as a tool for providing rehabilitation interventions at a distance (telerehabilitation). ^17^^,^^32^^,^^35^^–^^44^ Despite different designs and aims, most of the studies evaluated the effectiveness of the smartphone app use in telerehabilitation on pain and disability. Dario *et al.*, performed a systematic review with meta-analysis to evaluate the effectiveness of the intervention based on any form of telerehabilitation, phone calls, emailing, and web-based chats but also smartphone apps. On the basis of data from 11 included studies, the authors concluded moderate evidence that current telerehabilitation interventions are not more effective than minimal interventions on pain and disability outcomes. Their study also revealed that the effectiveness of such interventions remains understudied. ^40^ Indeed, only three RCTs evaluated the effectiveness of the smartphone apps use to provide telerehabilitation in LBP patients. Hou *et al.*, evaluated a system of telerehabilitation based on a smartphone app interface for patients after lumbar surgery for LBP and a web-based interface for physicians providing and surveying rehabilitation and communicating with the patient. The authors demonstrated that the intervention was more effective than usual care regarding disability and pain status (Ostwestry Disability Index, pain visual analogue scale) at 24-month follow-up. ^38^ Mbada *et al.*, in their RCT, compared telerehabilitation-based McKenzie therapy *versus* the same treatment but provided in the traditional face-to-face setting. The smartphone app interface was used to introduce the treatment and was supported by phone calls and text messages in the experimental group. In this study, clinical improvement was noted at 4- and 8-week follow-ups, but no difference was found between the two groups in pain intensity, back muscle endurance and activity limitation. Thus, the authors concluded that smartphone app-based McKenzie telerehabilitation can be successfully used especially to bridge the gap in the non-availability of clinic-based therapy. ^32^ Toelle *et al.*, investigated the effect of a multidisciplinary smartphone app ( *Kaia App*) on pain intensity at 12-week follow-ups in an RCT design. The experimental group received multidisciplinary self-management treatment based on current guidelines and supported by a chat with a healthcare professional *via* the app. The experimental group showed significantly lower pain intensity than the group receiving physiotherapy with on-line education. ^41^ The *Kaia App* was evaluated previously in a retrospective cohort study and showed good effect on pain intensity reduction, ^43^ and the analysis of the updated version of *Kaia App* revealed improvement in treatment adherence. ^42^ One more pilot RCT confirmed the feasibility and preliminary effectiveness of a physical activity intervention supported by a smartphone app on pain and disability. At the same time, patient acceptance and reduced care-seeking were observed. ^36^ The protocol of this study is detailed in a separate article. ^45^ Other studies focused on qualitative analysis of the patient’s preferences and compliance in the smartphone app use ^35^^,^^39^ and case series analysis of feasibility, effectiveness on pain and exercices adherence, and patient satisfaction with the telerehabilitation booster session. ^37^ Selter *et al.*, in their pilot study, assessed the validity of an image-based quantification of pain-related disability as well as patient compliance and patient-perceived utility of the smartphone app ( *Limbr*). ^44^ The results showed good compliance and patient-perceived utility. In addition, the authors noted good validity of an image-based quantification of pain-related disability. ^44^

### Use of smartphone apps for self-management

The most-studied use of the smartphone app in LBP patients was for self-management. We identified 29 articles focused on self-management of LBP *via* a smartphone app: four systematic reviews, ^10^^,^^33^^,^^46^^,^^47^ five RCTs, ^11^^,^^38^^,^^41^^,^^48^^,^^49^ five study protocols, ^18^^,^^45^^,^^50^^–^^52^ six qualitative studies, ^35^^,^^39^^,^^53^^–^^56^ three retrospective cohort studies, ^26^^,^^42^^,^^43^ five pilot RCTs, ^36^^,^^44^^,^^57^^,^^58^ and one case series. ^37^ Many of the studies coupled the self-management and telerehabilitation in the same app or in the same study intervention. These studies were presented in the previous section. ^32^^,^^35^^–^^45^ In a scoping review of the outcome used in the smartphone apps for self-management of LBP, Coe-O’Brien *et al.*, found 74 apps; only four used the outcome measure that could be linked to the International Classification of Functioning, Disability and Health System (ICF) core set for LBP. Furthermore, they concluded that most of the apps were of low quality, indicating the lack of outcome evaluation in the apps. ^33^ Machado *et al.*, performed a systematic review of smartphone apps for self-management of LBP. Similarly, they analysed 61 apps and found overall low quality of the apps, pointing to the lack of studies evaluating their effectiveness, presentation of the questionable information, and unattractive layouts. They also recommend that app developers collaborate with healthcare professionals and researchers to ensure the benefit for LBP patients. ^10^ A recent systematic review with meta-analysis analysed eHealth based self-management for chronic LBP. The term eHealth encompassed the interventions based on smartphone apps (m-Health) or on traditional Internet (web-Health) use, so studies included in this meta-analysis were not all based on smartphone apps. Nevertheless, the authors concluded low to moderate evidence of a positive impact on pain and disability of this type of self-management. ^46^ Previously Nicholl *et al.*, performed a systematic review of digital support interventions for the self-management of LBP. They revealed heterogeneity and low quality of the studies, which could not support the utility of digital based interventions for LBP. ^47^

A summary of the RCTs focused on effectiveness of smartphone apps targeting self-management for LBP is presented in Table 2 as is the quality evaluation using the PEDRO score. Chhabra *et al.*, evaluated the effect of the self-management app *Snapcare.* A control group of LBP patients received a written prescription of medication and home exercises and were compared to a self-management group focusing on physical activity improvement based on *Snapcare.* The results at 12 weeks showed a similar improvement in pain in both groups and greater improvement in function in the *Snapcare* than control group. The authors concluded that such results support the utility of *Snapcare* for LBP patients. ^11^ Suman *et al.*, performed a cluster RCT evaluating the effectiveness and cost-utility of a multifaceted eHealth intervention based on websites and social media platforms but also including a mobile version, adaptable to a smartphone. The intervention was inspired by the Australian mass media campaign promoting physical activity, positive back beliefs and coping with LBP. The control group received a digital patient information letter. The presented eHealth strategy was not effective in improving patients’ back pain beliefs or decreasing disability or absenteeism, but the study provided a favourable cost-utility analysis. ^48^ Irvine *et al.*, showed that their mobile app *FitBack* was effective in improving physical, behavioural and worksite outcomes. *FitBack* was based on a self-tailored cognitive-behavioural approach and used the American Pain Society recommendations. ^49^ Almhdawi *et al.*, also reported that use of their app called *Relieve my back* is efficient in pain and disability self-management. ^58^

**Table 2.** Summary of randomized controlled trials evaluating smartphone app-based self-management interventions for patients with low back pain (LBP).

Author, date (reference number)	Title	N of subjects who completed the study	Objective	Main outcomes and follow-up time	Results	Authors’ conclusion	PEDro score
Almhdawi, 2020 ^58^	Effectiveness of an innovative smartphone application for office workers with chronic non-specific LBP: a pilot RCT	n=40, EG, CG.	To evaluate the effectiveness of a newly developed evidence-based LBP management smartphone application.	Pain VAS, ODI, SF-12 at 6 weeks follow-up.	Significant pain improvement in EG group *vs.* CG (-3.45 (2.21) *vs.* -0.11 (1.66), p<0.001), in ODI score (-11.05 (10.40) *vs.* -0.58 (9.0), p=0.002), Significant increase in physical component of SF-12 (12.85 (17.20) *vs.* -4.63 (12.04), p=0.001).	‘ *Relieve my back*’ app reduce pain and disability and improve the quality of life of office workers with non-specific LBP.	9/10
Mbada, 2019 ^32^	Comparative effectiveness of clinic-based and Telerehabilitation Application of Mckenzie Therapy in chronic LBP.	CBMT group (n=26), TBMT group (n=21).	Compare the effects of TBMT and CBMT among patients with LBP.	PI, BEME, AL, PR, and GHS evaluated at 4 and 8 weeks.	Both groups improved significantly on PI, BEME, AL, PR and GHS (all p=0.001); no significant differences in treatment effects between TBMT and CBMT.	Smartphone-app based McKenzie therapy has comparable clinical effect with the traditional CBMT.	7/10
Hou, 2019 ^38^	The effectiveness and safety of utilizing mobile phone-based programs for rehabilitation after lumbar spinal surgery: Multicentre, prospective RCT.	Mobile phone-based EH group (n=84), UC treatment group (n=84).	The effectiveness of mobile phone-based rehabilitation systems in patients who underwent lumbar spinal surgery.	ODI, pain VAS, at baseline, 3, 6, 12, and 24 months postoperatively.	EH group present better ODI than UC group ODI at 24 months postoperatively, no significant difference in primary outcomes at other time points.	Mobile phone-based telerehabilitation program is effective in postoperative self-managed for patients after LBP surgery. This was more evident in participants with higher compliance.	7/10
Amorim, 2019 ^36^	Integrating Mobile-health, health coaching, and physical activity to reduce the burden of chronic LBP trial (IMPACT): a pilot RCT.	Intervention group (n=31), control group (n=24)	Investigate the feasibility and preliminary effectiveness of a patient-centred physical activity intervention, supported by health coaching and mobile health, to reduce care-seeking, pain and disability in patients with chronic LBP.	Care-seeking, pain levels and activity limitation evaluated at baseline, 6-month and weekly for 6 months.	Care seeking reduction in intervention group (38%). No between-group differences for pain levels or activity limitation.	Mobile-health coaching physical activity approach was feasible and well accepted by participants and may reduce care-seeking in patients with LBP. Further study with better statistical power is needed.	8/10
Chhabra, 2018 ^11^	Smartphone app in self-management of chronic LBP: an RCT.	App group (n=45), control group (n=48).	The purpose of this study was to examine the effect of using a smartphone app (called *Snapcare*) on pain and function in patients with chronic LBP.	NPRS and MODI, evaluated at baseline and after 12 weeks of treatment.	Both groups improved in NPRS and MODI (p<0.05). The app group showed a significantly greater improvement in MODI (p<0.001).	*Snapcare* app facilitated an increase in physical activity and improved pain and disability in patients with chronic LBP.	7/10
Yang, 2019 ^57^	Smartphone-based remote self-management of chronic LBP: a preliminary study.	Self-management + physiotherapy group (n=5), control group physiotherapy only (n=3).	To assess the additional effect of self-management on physiotherapy by the use of apps on management of chronic LBP.	Pain VAS, PSEQ, RMDQ, and SF36. Baseline, week 2, and week 4 posttreatment.	Experimental group was better in PSEQ (p=0.035), RMDQ (p=0.035), SF36-Bodily Pain (p=0.008), and SF36-Mental Health (p=0.013).	Smartphone app-based self-management program gives additional benefits to physiotherapy for patients with chronic LBP.	5/10
Suman, 2019 ^48^	Effectiveness and cost-utility of a multifaceted eHealth strategy to improve back pain beliefs of patients with non-specific LBP: a cluster randomised trial.	Intervention group (n=200), control group (n=347).	To assess the effectiveness and cost-utility of a multifaceted eHealth strategy (including mobile version) compared to usual care in improving patients' back pain beliefs, and in decreasing disability and absenteeism.	BBQ, RDQ-24, PRODISQ, and TIC-P at baseline, 3, 6 and 12 months.	No differences between groups in back pain beliefs, disability, or absenteeism. Mean intervention costs were in favour of the intervention group (EUR 70, and the societal cost difference EUR 535), but no significant cost savings were found. The ICER showed that the intervention dominated usual care and the probability of cost-effectiveness was 0.85 on a willingness-to-pay of EUR 10.000/QALY.	Promising cost-utility results based on QALYs are in favour of eHealth strategy. However, it was not accompanied by improving patients' back pain beliefs or decreasing disability and absenteeism.	8/10
Toelle, 2019 ^41^	App-based multidisciplinary back pain treatment vs. combined physiotherapy plus online education: an RCT.	*Kaia App* group (n=42), control group (n=44).	To investigate the clinical effects of a multidisciplinary mHealth back pain app ( *Kaia App*) in an RCT.	NRS for pain intensity, at 12-week.	*Kaia App* group reported significantly better pain improvement than the control group (mean 2.70 [1.51] *vs.* 3.40 [1.63]).	App-based multidisciplinary back pain treatment is effective for LBP patients and is better than physiotherapy combined with online education.	5/10

RCT, randomized controlled trail; LBP, low back pain; EG, experimental group; CG, control group; VAS, visual analogue scale; ODI, Ostwestry Disability Index; SF-12, Short-Form Health Survey; CBMT, Clinic-Based McKenzie Therapy; TBMT, Telerehabilitation-Based McKenzie Therapy; PI, pain intensity; BEME, back extensor muscle endurance; AL, activity limitation; PR, participation restriction; GSH, general health status; EH, eHealth; UC, usual care; NPRS, Numeric Pain Rating Scale; MODI, Modified Oswestry Disability Index; PSEQ, Pain Self-Efficacy Questionnaire; RMDQ, Roland Morris Disability Questionnaire; BBQ, Back Beliefs Questionnaire; RDQ-24, Roland Disability Questionnaire; PRODISQ, Productivity and Disease Questionnaire; TIC-P, Trimbos/iMTA questionnaire for Costs associated with Psychiatric Illness; ICER, Incremental cost-effectiveness ratio; QALY, quality-adjusted life years; NRS, Numeric rating scale.

We also found several articles presenting study protocols for smartphone apps used by LBP patients. Sandal *et al.*, presented the protocol of an RCT comparing the effect of usual care supported by the *selfBACK* app *versus* usual care only. Tailored self-management plans were provided by the *selfBACK* app consisting of advice on physical activity, physical exercises, and educational content. Self-management plans were prepared by using case-based reasoning methodology, a branch of artificial intelligence. ^50^ Mork *et al.*, presented complementary information regarding the implementation of the methodology used in the *selfBACK* protocol. ^18^ One RCT protocol aimed to evaluate the effectiveness of *Relaxback*, focusing on relaxation for LBP patients in reducing pain. Autogenic training, mindfulness meditation and guided imagery is used in the app and will be compared to usual care. ^51^ Another RCT protocol is for *MIMate*, designed to support motivational interviewing performed by a physical therapist and targeting behavioural changes regarding physical activity. *MIMate* is used between face-to-face physical therapy sessions and is compared to usual physical therapy sessions. ^52^

Two qualitative studies coupled telerehabilitation and self-management interventions and were mentioned in the previous section. ^32^^,^^36^ Nevertheless, four other qualitative studies investigated the preference in content of an app improving self-management ^53^^,^^55^ or the feasibility/utility of an app providing educational videos focused on self-management, postoperative protocols, or tailored self-management plans. ^54^^,^^56^ Three retrospective cohort studies using smartphone apps for self-management of LBP were coupled with telerehabilitation, evaluation or data collection and were mentioned in the previous sections. ^26^^,^^42^^,^^43^ Similarly, in two pilot studies, self-management was coupled with telerehabilitation; these articles were presented in the previous section. ^36^^,^^44^ However, another pilot RCT, used a smartphone app to enhance self-management between physical therapy sessions and compared it to physical therapy only. The *Pain Care* app provided self-management based on home exercises. The authors concluded that a more powerful study needed to be conducted considering their promising results. ^57^

### Use of smartphone apps for data collection

Some studies used smartphone apps for LBP patients to collect medical information. One study protocol presented a smartphone app to collect timely data in ecological situations, including pain intensity, physical function, analgesic use and adherence to auricular point acupressure treatment. ^59^ Similarly, in a study that aimed to evaluate relations between opioid use and pain intensity, a smartphone app was used exclusively to collect information about pain intensity multiple times daily. ^60^ However, in most studies, data were collected together with other uses of the smartphone app previously presented: self-management, telerehabilitation, evaluation. ^23^^–^^26^^,^^42^^,^^43^^,^^50^^,^^54^

## Discussion

The aim of this scoping review was to identify the current fields of employment of smartphone apps for LBP patients. Although the use of smartphone apps for LBP patients is relatively recent, the scope of our review appears to be large, with 43 articles meeting our inclusion criteria. Emerging uses of the smartphone apps are self-management, telerehabilitation, evaluation and data collection. The present review did not aim to firmly classify use of the smartphone apps, as their multiple uses can be controversial. Rather we aimed at investigating what could be the utility of the apps for LBP patients, the current scientific knowledge and perspectives that are worthy of study and development.

We observed a notable interest in scientific literature regarding the utilization of smartphone apps for patients with LBP. Self-management is a field of smartphone app use that has gained the most attention. Telerehabilitation is often coupled with self-management, and data collection is usually integrated with evaluation. The evidence of effectiveness of smartphone apps in self-management of LBP derived from RCTs are favourable on pain and disability for patients with chronic LBP ^11^^,^^32^^,^^41^^,^^49^^,^^57^ and patients after spinal surgery for LBP. ^38^ However, one RCT reported no effect of such interventions on pain, disability and beliefs of LBP patients but showed promising cost-utility results. ^48^

Overall, the quality of the analysed RCTs was moderate to good as assessed by the PEDro score. Indeed, the content of the interventions varied between the studies, and different apps presented differences in providing self-management. Nevertheless, improving physical activity level and providing education about LBP were the common components of the interventions. Self-management with a smartphone app was frequently used together with other interventions including face-to-face physical therapy, web-based education or email reminders. The systematic reviews focused on self-management actually reviewed app stores to find existing self-management apps rather than screening the scientific literature data ^10^^,^^33^ or included the studies focused more largely on eHealth interventions. ^46^^,^^47^ The effectiveness of the telerehabilitation interventions including smartphone apps evaluated by Dario *et al.*, in a 2017 systematic review showed moderate evidence that telerehabilitation is not more effective than minimal interventions for pain and disability outcomes. ^40^ However, since then, new RTCs have shown more optimistic results. ^32^^,^^38^^,^^41^ Telerehabilitation and self-management of LBP are in a phase of dynamic development; possibly favourable results in recent studies correspond to improvements in providing such interventions.

Smartphone apps used as an evaluation tool showed good measurement proprieties for pain evaluation ^23^^–^^26^ and contradictory for spinal ROM measurements. ^16^^,^^27^^–^^29^ Indeed, technical aspects of such measurement are complicated: controlling all potential error sources in a multi-segmental movement is challenging. This observation is consistent with other spinal ROM measurement studies also indicating contradictory results. ^61^^,^^62^ Using the therapist's personal smartphone to perform measurements on patients may not be practical. However, remote clinical evaluation using a smartphone app should be developed regarding the consistent results between remote and face-to-face subgrouping of patients by treatment-based classification, the McKenzie system and the Backache Disability Index. ^17^^,^^32^^,^^34^

Use of smartphone apps for data collection seems highly useful and effective. Smartphones are personal and easily accessible to collect data. Many of the studies we found used smartphone apps to collect information, even if the main use was self-management, telerehabilitation, or evaluation. It seems pertinent to develop this branch of smartphone use. At the same time, it's important to respect data security considerations. Yet, our review also reveals development in data treatment. A recently started study implements machine learning technology to provide a personal adapted self-management strategy. ^50^ For this form of analysis, a large amount of data needs to be collected and smartphones perfectly fit this goal.

### Limitations

Some articles could have been missed in our search strategy as we only used three databases. However, based on a large problem of our review, identifying the current fields of the smartphone apps use for LBP patients’ points out perspectives for further studies including perspectives for a more specific systematic review. Also, the highly heterogenic terminology regarding smartphone apps use could have resulted in some omissions. Several studies using eHealth strategy ( *e.g.*, tablet apps) were not included if there was no clear information about the smartphone app use.

## Conclusions

The present scoping review revealed that the scientific literature interest in the utilization of smartphone apps for patients with LBP is notable. The main uses are for self-management, telerehabilitation, evaluation and data collection. Self-management is the most used in LBP and showed moderate- to good-quality evidence for effectiveness on pain and disability. Regarding technological and socio-cultural development, new fields of use may arise.

## Data availability

### Underlying data

All data underlying the results are available as part of the article and no additional source data are required.

## Reporting guidelines

Figshare: PRISMA-ScR checklist for ‘Smartphone applications are used for self-management, telerehabilitation, evaluation and data collection in low back pain healthcare: a scoping review’, https://doi.org/10.6084/m9.figshare.20555802.v1. ^63^

Data are available under the terms of the Creative Commons Zero “No rights reserved” data waiver (CC0 1.0 Public domain dedication).
