# Supplementary material for: Smartphone applications are used for self-management, telerehabilitation, evaluation and data collection in low back pain healthcare: a scoping review
Source: F1000Res. 2024 May 7;11:1001. Originally published 2022 Sep 6. [Version 2] doi: 10.12688/f1000research.123331.2 (PMC11153999; doi:10.12688/f1000research.123331.2)
Supplement: Peer review history

# Reviewer report: Reviewer response for version 2

**DOI:** 10.5256/f1000research.164960.r274858

**Published:** 2024-06-05

**Authors:** Marijn Ulrich

**Affiliations:** 1 Utrecht University of Applied Science, Utrecht, The Netherlands

I agree with all of the changes you made in the article. Also with the improvements you made which did not directly address my concerns.

 I have no further concerns about your article. It has become a well written and interesting contribution to this field of research.

Are the rationale for, and objectives of, the Systematic Review clearly stated?

Yes

Is the statistical analysis and its interpretation appropriate?

Not applicable

Are sufficient details of the methods and analysis provided to allow replication by others?

Partly

Are the conclusions drawn adequately supported by the results presented in the review?

Partly

Reviewer Expertise:

My are of research is implementation of smartphone apps, or digital health, in the context of physiotherapy for people with low back pain.

I confirm that I have read this submission and believe that I have an appropriate level of expertise to confirm that it is of an acceptable scientific standard.

# Reviewer report: Reviewer response for version 2

**DOI:** 10.5256/f1000research.164960.r274859

**Published:** 2024-05-09

**Authors:** Alexandra Roren

**Affiliations:** 1 INSERM UMR-S 1153, Service de Rééducation et de Réadaptation de l'Appareil Locomoteur et des Pathologies du Rachis, PRES Sorbonne Paris Cité, ECaMO team, Institut Fédératif de Recherche sur le Handicap, Hôpital Cochin, AP-HP, Université Paris Descartes, Paris, France

I approve  this revised version. No further comments to make.

Are the rationale for, and objectives of, the Systematic Review clearly stated?

Yes

Is the statistical analysis and its interpretation appropriate?

Not applicable

Are sufficient details of the methods and analysis provided to allow replication by others?

Yes

Are the conclusions drawn adequately supported by the results presented in the review?

Yes

Reviewer Expertise:

Shoulder and trunk kinematics, development and assessment of innovative tools to assess and treat people whith chronic musculoskeletic disease, adhesion to treatment based on physical activity.

I confirm that I have read this submission and believe that I have an appropriate level of expertise to confirm that it is of an acceptable scientific standard.

# Reviewer report: Reviewer response for version 1

**DOI:** 10.5256/f1000research.135427.r253130

**Published:** 2024-03-28

**Authors:** Marijn Ulrich

**Affiliations:** 1 Utrecht University of Applied Science, Utrecht, The Netherlands

First of all, I would like to complement the authors on this clearly written and relevant scoping review. It's nice to read a review with a broader scope than just evidence of treatment effect.

 The rationale and objectives of the study are clear for me. The introduction gives good context on the topic.

 The methods used are quite clear and repeatable, but with some remarks:

 - In the exclusion criteria, it's not fully clear if you also excluded physical activity promotion or lifestyle advise for people with low back pain (or only for healthy people).

 - You included study protocols, but what did you do when those studies were performed and published? Since they can describe the exact same study, can you mention if you excluded protocols when the article of the finished study was published for example. Or any other way of handling them if you included them both.

 - In the synthesis, can you explain how you drew conclusions like 'promising'. Did you draw those conclusions yourselves or did you take (copy-paste) these conclusions from the included article (like you do mention in table 2 under 'author's conclusion')?

 This last point applies for the results and discussion/conclusion as well. Quite often you use conclusions like 'promising'. To me, it's too subjective (and perhaps biased). It would be nice if you can give some clarity on that. I understand it's not the main goal like in a systematic review or meta-analysis.

Are the rationale for, and objectives of, the Systematic Review clearly stated?

Yes

Is the statistical analysis and its interpretation appropriate?

Not applicable

Are sufficient details of the methods and analysis provided to allow replication by others?

Partly

Are the conclusions drawn adequately supported by the results presented in the review?

Partly

Reviewer Expertise:

My are of research is implementation of smartphone apps, or digital health, in the context of physiotherapy for people with low back pain.

I confirm that I have read this submission and believe that I have an appropriate level of expertise to confirm that it is of an acceptable scientific standard, however I have significant reservations, as outlined above.

# Reviewer report: Reviewer response for version 1

**DOI:** 10.5256/f1000research.135427.r152995

**Published:** 2022-10-24

**Authors:** Alexandra Roren

**Affiliations:** 1 INSERM UMR-S 1153, Service de Rééducation et de Réadaptation de l'Appareil Locomoteur et des Pathologies du Rachis, PRES Sorbonne Paris Cité, ECaMO team, Institut Fédératif de Recherche sur le Handicap, Hôpital Cochin, AP-HP, Université Paris Descartes, Paris, France

This scoping review is well written. It's an interesting work that takes stock of a current topic. 

 My minor remarks are the following:

**Abstract: **

- Background - There is a gap between the first (too general) and second sentences. 
- Conclusions - You can't state the growing literature as you didn't really study this aspect (quantitative assessment of studies through the years).

**Introduction:**

- First paragraph: Are "Navigation, video communication, gaming, and social media" the most common use of mobile phones?
- Third paragraph: You should make a difference between LBP and chronic LBP which is in some conditions the major target of biopsychosocial management.
- Fourth paragraph, first sentence: Does the "purpose" include overtreating?
- You could also raise the topic of the cost and accessibility of the apps compared to usual (face-to-face) treatment.

**Methods**

- Could you justify why you included studies from January 1st, 2005?

**Results:**

- Use of smartphone apps for evaluation: Give a few words about PainMAP.
- When you talk about spine surgery, specify that you are talking of "spine surgery" for low back pain. 

**Discussion:**

- First paragraph: To explain why the classification could be controversial, you could specify that the use of apps is multiple. 
- It's not clear why you use both the words "efficacy" and "effectiveness".
- Second paragraph: As stated above, you can't really state about the growing literature as you didn't really study that point.
- When you are talking of efficacy or effectiveness, specify systematically on what outcomes.
- I don't really understand the problem of hygiene raised by the use of apps to measure RoM. 
- Last paragraph before the limits part, what about data security?

**Limitations:**

- I'm not sure that the compulsive behavior is a limitation as it is not really in the scope of your review.

**Conclusions:**

- Same remark about "growing literature".

Are the rationale for, and objectives of, the Systematic Review clearly stated?

Yes

Is the statistical analysis and its interpretation appropriate?

Not applicable

Are sufficient details of the methods and analysis provided to allow replication by others?

Yes

Are the conclusions drawn adequately supported by the results presented in the review?

Yes

Reviewer Expertise:

Shoulder and trunk kinematics, development and assessment of innovative tools to assess and treat people whith chronic musculoskeletic disease, adhesion to treatment based on physical activity.

I confirm that I have read this submission and believe that I have an appropriate level of expertise to confirm that it is of an acceptable scientific standard.
